# Croup

**Published:** 1874-06

**Authors:** 


### CROUP

causes death by suffocation. The entrance to the windpipe is very small; a little cold causes the lining of the part called the mucous membrane to swell. This diminishes the opening, which is made smaller still by what is called sub-mucous infiltration—that is, this mucous membrane being inflamed, throws out an extra amount of fluid, like the eye, when it is inflamed. This fluid hardens and forms at length a kind of layer, which is sometimes of an almost leathery toughness, increasing in thickness until the orifice is so nearly closed that the breath is obstructed. Nauseating medicines dilute this formation and thus aid to bring it away.

A favorite prescription for a quarter of a century, with eminent physicians, was to mix a teaspoonful of powdered alum with a little sugar to make it palatable.

The immediate effect is to nauseate and vomit, giving great relief, in a minute, sometimes.

Flannels dipped in ice cold water, changed every two minutes, and squeezed a little, so as not to dribble and wet the clothing, is an excellent remedy, because it cools the parts and thus diminishes the amount of blood sent there, and as the phlegm is made out of the blood, a less amount is made, and relief is certain. But flannels dipped in water as hot as can be borne and applied to the part, changed every two minutes, carries off the heat by evaporation, and irritating the surface brings the blood away from the interior and thus diminishes the phlegm.

